# Coaching to strengthen critical success factors in integrative care for chronic fatigue patients: the Patient Needs-Resources Model

**DOI:** 10.3389/fnins.2023.1202930

**Published:** 2023-07-21

**Authors:** Diana Araja, Angelika Krumina, Zaiga Nora-Krukle, Marion E. Schneider, Uldis Berkis, Modra Murovska

**Affiliations:** ^1^Institute of Microbiology and Virology, Riga Stradins University, Riga, Latvia; ^2^Department of Infectology, Riga Stradins University, Riga, Latvia; ^3^Department of Experimental Anesthesiology, University of Ulm, Ulm, Germany; ^4^Development and Project Department, Riga Stradins University, Riga, Latvia

**Keywords:** myalgic encephalomyelitis/chronic fatigue syndrome (ME/CFS), holistic approach, multidisciplinary team, patient engagement, health-related quality of life (HRQoL)

## Abstract

Theoretical and empirical studies discover that an integrative approach is particularly important in chronic disorders and multiple long-term conditions, such as chronic fatigue. Chronic fatigue syndrome (CFS) is a classic example of a potentially severe, multisystemic illness with a wide diversity of symptoms and the corresponding diagnostic complexity. The prevalence of CFS-like syndromes expanded in the context of the COVID-19 pandemic, increasing the disorder and treatment burden. Thus, this article aimed to draw attention to the possibilities to strengthen the integrative approach to diagnosing and treating chronic disorders and multiple long-term conditions. The main critical success factors identified for integrative approaches were: a holistic approach, that provides a more comprehensive diagnostic and personalized treatment strategy, a multidisciplinary team, and patient engagement. The strengths and weaknesses of these factors were explored and coaching was identified as a potential unifying and reinforcing element. Coaching has a wide spectrum of manifestations clearly representing a holistic approach, that has been successfully used in multidisciplinary team building. Moreover, coaching exposes support addressing the patient engagement issues identified by the Patient Needs-Resources Model (PN-R Model) such as low levels of self-efficacy, optimism, and subjective well-being. Coaching may assist patients to identify and prioritize their goals, becoming aware of their personal resources, developing strategies for managing symptoms, and building skills to increase their self-efficacy and active engagement in the treatment process. Therefore, the authors emphasize coaching as a perspective element of optimization of patient care, that requires additional theoretical and long-term empirical research.

## Introduction

Chronic fatigue is manifested in different perspectives – as a complex disorder such as chronic fatigue syndrome (CFS) or myalgic encephalomyelitis/chronic fatigue syndrome (ME/CFS), or chronic fatigue as a medical condition accompanying other disorders. CFS is a heterogenous disorder of multiple disabling symptoms with complex manifestations in which patients experience a wide range of symptoms that are broadly categorized as neurological, immunological, autonomic and endocrinological (Kujawski et al., 2021). Principal component analyses, utilized to identify symptom subgroups and the relationships with functioning and quality of life, suggested four statistically distinct and clinically meaningful subgroups of symptoms: inflammatory, pain, neurocognitive, and autonomic; all symptom subgroups correlated significantly with measures of fatigue, mood, functioning and quality of life (Jonsjö et al., 2017).

CFS is also characterized by neuro-psychiatric (e.g., depression, irritability, sleep disorders, autonomic symptoms and neurocognitive defects) and physio-somatic (fatigue, a flu-like malaise, hyperalgesia, irritable bowel, muscle pain and tension) symptoms (Maes, 2015). According to a review of case definitions, at least 25 case definitions/diagnostic criteria based on three conceptual factors (aetiology, pathophysiology, and exclusionary disorders) were developed between 1986 and 2020 (Lim and Son, 2020).

Regarding prevalence, ME/CFS affects 0.4% of the population, 25% of which experience the severe and very severe categories; these are defined as being wheelchair-, house-, and bed-bound (Strassheim et al., 2021). CFS spectrum is expanded as a result of the disease caused by the SARS-CoV-2 virus (COVID-19) pandemic and as a concomitant element of long-COVID (Araja et al., 2021; Davis et al., 2021; Friedman et al., 2021; Gaber, 2021; Graham et al., 2021; Jason et al., 2021; Komaroff and Bateman, 2021; Komaroff and Lipkin, 2021; Paul et al., 2021; Poenaru et al., 2021; Simani et al., 2021; Townsend et al., 2021; Wong and Weitzer, 2021; Araja et al., 2022). At the same time, the financial burden increases accordingly. Prior to the COVID-19 pandemic, researchers estimated a United States (U.S.) ME/CFS prevalence of 1.5 million and an annual economic impact of $36–51 billion (Mirin et al., 2022). In 2022, due to the COVID-19 pandemic and its resulting post-acute sequalae, it was estimated that total ME/CFS prevalence could rise to between five and nine million. This would incur an annual U.S. economic impact of $149 to $362 billion in medical expenses and lost income, exclusive of other costs, such as disability benefits, social services, and lost wages of caretakers (Mirin et al., 2022).

Due to difficulties in diagnosing and treating CFS (or ME/CFS), its different manifestations, and its severity, patients are exposed to a significant burden of the disorder. At the same time, the humanistic or patient-centric view argues strongly that populations with physical, developmental or cognitive disabilities – often with related chronic conditions or complex illnesses – endow the concept of healthcare integration with a unique logic and meaning (Kodner and Spreeuwenberg, 2002). Vulnerable individuals, such as the diverse group described above, have complicated and ongoing needs (which frequently are part-medical, part-physical, part-psychological, and part-social), experience difficulties in everyday living, require a mix of services delivered sequentially or simultaneously by multiple providers and receive both cure and care in the home, community and institutional settings (Kodner and Spreeuwenberg, 2002).

Therefore, the authors considered that CFS is a classic example of a disorder in which integrative care should play a key role in the choice and implementation of diagnostic, treatment and care pathways. However, given the slow uptake of this approach in practice, the authors posed a research question within this perspective: what are the critical success factors of an integrative approach in chronic disorders and multiple long-term conditions and how can these be strengthened? Accordingly, this article aimed to explore the possibilities to strengthen the integrative care approach.

After reviewing the theoretical background and practical considerations, the authors came to the intermediate conclusion that a holistic approach, a multidisciplinary team, and patient engagement are the critical success factors for an integrative approach. In order to identify phenomena that could strengthen this triad, the traits of their development were assessed and a potentially strengthening element was identified – coaching, which is characterized by the holistic approach, is applicable for promoting team cohesion and strengthening patients’ personal resources. Coaching, which includes working with a trained professional to set goals, develop strategies, and build skills, has the potential to encourage a holistic approach and improve multidisciplinary team building and patient engagement in the management of chronic disorders such as CFS.

## Integrative care for patients with chronic disorders: critical success factors

A contemporary multidisciplinary stakeholder-informed definition of integrative healthcare is generated by Leach et al. (2018), based on a thematic analysis of data drawn from healthcare consumers/providers, integrative healthcare organization webpages, and eligible articles. The consensus is reached on a single definition of integrative healthcare: “Integrative healthcare is a collaborative, coordinated, transdisciplinary, person-centered model of care informed by a holistic model of health and the best available evidence; care is facilitated by an interdependent, multi-disciplinary team of like-minded, biomedical, allied and complementary health professionals that work together in a collegial, non-hierarchical, communicative and respectful environment in order to prevent illness and optimize health, healing, and wellness in individual clients” (Leach et al., 2018).

This definition includes a number of conceptual notions, and each of them is worth exploring in more depth, including the client-centered approach which is not a new concept – it dates back to the 1940s when Rogers described non-directive client-centered therapy (Rogers, 1946). Unlike other therapies in which the skills of the therapist are to be exercised upon the client, in this approach the skills of the therapist are focused on creating a psychological atmosphere in which the client can work with his/her own residing constructive forces whose strength and uniformity have been either entirely unrecognized or grossly underestimated (Rogers, 1946).

Later, in the 1950–1960s, Balint reinforced this approach and introduced the term “patient-centered medicine” which called for examining patients’ psychological needs in addition to their biological symptoms and for a view of the patient as a unique human being, in contrast to “illness-oriented medicine” (Balint, 1955; Balint, 1969). The illness-oriented biomedical model represents the application to the medicine of the classical factor-analytic approach, focuses on the disease, and does not consider patients’ experience of their illness or how their social environment and circumstances affect how they view their illness (Engel, 1980).

In the 1970–1980s, Engel introduced the biopsychosocial model as a scientific model constructed to take into account the missing dimensions of the biomedical model and to promote the holistic health view (Engel, 1980). Since patient-centered approach’s inception, many models and definitions have been proposed in the literature (Gerteis et al., 1993; Mead and Bower, 2000; Stewart et al., 2000; Davis et al., 2005; Epstein et al., 2005; Clayton et al., 2011; Barry and Edgman-Levitan, 2012; Fredericks et al., 2012; Van Berckelaer et al., 2012; Kitson et al., 2013; Stewart et al., 2013; Moreau et al., 2020), although their practical implementation is lagging behind the theory, as this approach requires significant core changes in the norms and expectations for most healthcare systems.

The results of examining the evolution of healthcare integration (Evans et al., 2013) through a review and synthesis of over 25 years of international academic research and literature, demonstrated six major, inter-related shifts in integration strategies: (a) from a focus on horizontal integration to an emphasis on vertical integration; (b) from acute care and institution-centered models of integration to a broader focus on community-based health and social services; (c) from economic arguments for integration to an emphasis on improving quality of care and creating value; (d) from evaluations of integration using an organizational perspective to an emerging interest in patient-centered measures; (e) from a focus on modifying organizational and environmental structures to an emphasis on changing ways of working and influencing underlying cultural attitudes and norms; and (f) from integration for all patients within defined regions to a strategic focus on integrating care for specific populations (Evans et al., 2013). Additionally, it was noted that many of these shifts are a growing recognition of the value of understanding healthcare integration as processes situated in Complex-Adaptive Systems (CAS; Evans et al., 2013). Assuming the healthcare system as a CAS provides additional opportunities for understanding a healthcare system’s functioning, governance, and decision-making (Araja, 2022).

Integrative care is represented mostly in the context of integrated care, which also has many definitions and interpretations that complicate its perception and implementation. There are multiple levels of integrated care, and four levels of health services delivery are investigated more often: the personal, the professional, the management, and the system level (Valentijn et al., 2013; Zonneveld et al., 2020) In addition to the different levels, researchers present two crucial dimensions of integration: systemic integration, which includes the coherence of rules and policies in the healthcare system, and normative integration, which comprises the role of shared values in coordination and collaboration (Valentijn et al., 2013; Valentijn, 2016; Zonneveld et al., 2020).

The integrative approach is most closely linked to the two levels of the integrated healthcare system: the personal (the interaction between patient and care provider) and the professional (the interaction between care providers). The research and thematic analysis performed by Leach et al. (2018) identified seven distinct themes on integrative healthcare, which could be refined into three interrelated and interdependent constructs, as the triad (critical success factors) of integrative healthcare – the client, the team, and the approach to care.

The authors formulated this triad in the framework of the current study as a holistic approach, multidisciplinary team, and patient engagement.

## Holistic approach, multidisciplinary team, and patient engagement models

The theoretical framework of the holistic approach is mainly based on Smuts’ concepts defined in the book “Holism and Evolution” (1926), among other insights declared, that “Personality as a whole, as a form, is indeed the highest form of Holism” (Smuts, 1926). Accordingly, holistic medicine concepts have evolved over time, but there remain contradictions with the dominant biomedical paradigm. Value-based healthcare implies that healthcare issues are addressed most effectively with the “physicians in the lead” strategy. However, according to diverse stakeholders, (Malik et al., 2018) “physicians in the lead” strategy does not support a holistic healthcare delivery approach, primarily because of the strong biomedical focus of the physicians. Although physicians can be educated to place more emphasis on holistic outcomes, holistic care delivery requires greater integration and teamwork in the care chain. As different healthcare professions are complementary to each other, a new strategy of a “team in the lead” was suggested to meet the holistic healthcare demands (Malik et al., 2018).

There are positive examples in the literature of multidisciplinary assessment of chronic fatigue, following a holistic approach that is based on the biopsychosocial model (Mariman et al., 2013), but a multidisciplinary team is more often seen in the diagnosis setting than in the treatment process. Also, some findings (Kvarnström, 2008) show difficulties related to the team dynamic that arose when team members acted toward one another as representatives of their professions, difficulties that occurred when the members’ various knowledge contributions interacted in the team, and difficulties related to the influence of the surrounding organization. The perceived consequences of the difficulties, beyond individual consequences, were restrictions on the use of collaborative resources to arrive at a holistic view of the patient’s problem, and barriers to providing patient care and service in the desired manner (Kvarnström, 2008).

Therefore, the authors propose to recognize multidisciplinary team building as a deliberately managed process in the context of human resources (HR) management and co-creation. HR co-creation is a continuous process in which HR and stakeholders optimize value through collaborative efforts to innovate in the design and use of HR practices to better satisfy multiple stakeholders’ needs (Hewett and Shantz, 2021). In the framework of interaction with stakeholders, it is useful to use already established models, such as the Harvard model, (Bondarouk and Brewster, 2016) that detail interactions with different stakeholders, which in the case of chronic diseases will include patients, families, care providers, patient organizations, local authorities, public institutions, scientific representatives and other stakeholders.

At the same time, the authors assume that the patient’s personal resources are the crucial aspect in the co-creation of disease management and recovery. Personal resources mainly include self-efficacy, optimism, and subjective well-being. According to Bandura, the self-efficacy portion of Social Cognitive Theory addresses the origin of self-efficacy beliefs, their structure and functional properties, their diverse effects, the processes through which they work, and how to develop and enlist such beliefs for personal and social change (Bandura, 1997). Optimism refers to the expectancy of a positive outcome that encourages persistence in the face of obstacles, and subjective well-being, in turn, refers to a psychological asset that helps individuals to carry out their objectives in the best psychological conditions (Neveu et al., 2023). The Conservation of Resources theory predicts that resource loss is the principal ingredient in the stress process (Hobfoll, 2001).

Therefore, the common trait of integrative approaches is that they require both a multidisciplinary team and the active engagement of patients in the treatment process. A concept analysis performed by Higgins et al. (2017) revealed four defining attributes of patient engagement: personalization, access, commitment, and therapeutic alliance. Patient engagement is defined as the desire and capability to actively choose to participate in care in a way uniquely appropriate to the individual, in cooperation with a healthcare provider or institution, for the purposes of maximizing outcomes or improving experiences of care (Higgins et al., 2017).

Researchers had proposed different models for patient engagement, one of the most well-known being the Patient Health Engagement Model (PHE Model), developed by Graffigna and colleagues (Graffigna et al., 2014). According to this model, the disease onset has a great influence on the patient’s psychological functioning, and a better balance is expected to be produced among the three experiential dimensions implied in the health management process: think (cognitive level), feel (emotional level), and act (behavioral level) (Graffigna et al., 2014). Other models such as the Patient-derived Model of Patient Engagement *via* Patient and Family Advisory Councils (PFACs; Dukhanin et al., 2020), the “Ottawa Model” for patient engagement in research (Vanderhout et al., 2022), and Pre-screening Models for Patient Engagement: the MOPEAD project (Boada et al., 2020) are also used. However, it should be noted that, despite the methodological basis, the practical patient engagement process is slowed.

## Discussion

The authors assume that current models do not adequately address the crucial aspect – the patient’s personal resources. It is therefore worth paying attention to HR models, such as the Job Demands-Resources (JD-R) model (Demerouti and Bakker, 2011). The JD-R model is a theoretical framework that tries to integrate two fairly independent research traditions: the stress research tradition and the motivation research tradition. According to the JD-R model, job demands are initiators of a health impairment process and job resources (social support, performance feedback, autonomy) are initiators of a motivational process (Demerouti and Bakker, 2011). However, the focus of this model is mostly on the relationship between job demand and job resources, not on personal resources.

**Figure 1:**
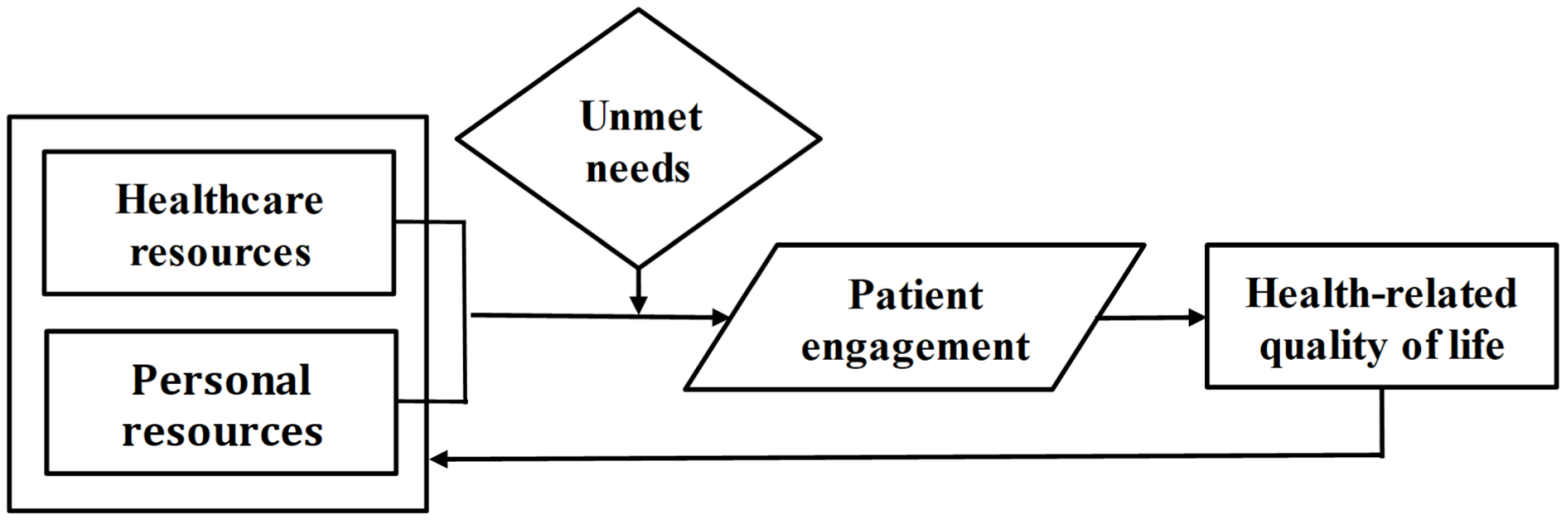
Conceptual framework of the proposed Patient Needs-Resources Model (PN-R Model).

Based on the theories observed, the authors propose an option of the Patient Needs-Resources Model (PN-R Model), the conceptual framework of which is shown in Figure 1.

The PN-R Model (Figure 1) suggests that Health-related quality of life (HRQoL) is the dependent variable; Healthcare resources and Personal resources are the independent variables. Accordingly, Patient engagement is a mediator, and Unmet needs – are a moderator. The impact of specific diseases on HRQoL might be taken into account, as for example, ME/CFS can reduce the score of HRQoL by an average of 40 points out of 100, measured by the Visual Analogue Scale (Brenna et al., 2021). Healthcare resources can be defined in the context of the World Health Organization’s healthcare system building blocks: service delivery, health workforce, information, medical technologies, financing, leadership and governance, which are used also in the evaluation of healthcare resilience (Fridell et al., 2020). Unmet medical needs may be related to the availability, accessibility, affordability, and acceptability of healthcare, assessed by Multi-criteria Decision Analysis, and specific needs related to a particular diagnosis or medical condition (Araja, 2018).

The PN-R Model might operate in such a way that, by using healthcare resources and personal resources and being aware of unmet needs, the patient actively engages in a treatment process that improves their HRQoL, which returns as enhanced personal resources. However, in real life, personal resources, which include self-efficacy, optimism, and subjective well-being, are the weakest point in ensuring patient engagement. It should be noted that a person needs energy, strength, and a clear mind to demonstrate self-efficacy, optimism, and subjective well-being, which most often patients do not have, especially chronic fatigue patients.

In this context, and in light of the authors’ performed recent research on the advantages of an integrative approach in the primary healthcare of post-COVID-19 and ME/CFS patients (Araja et al., 2023), the authors suggest that coaching is important in working with chronic fatigue patients. A case study analysis demonstrates significant improvements in the health status of ME/CFS and post-COVID-19 patients assessed by the EuroQol-5D-5L tool (Araja et al., 2023). Current evidence, produced by a systematic review and meta-analysis of randomized controlled trials, suggests also that health coaching could reduce blood pressure, improve dietary behaviors, and increase self-efficacy and awareness among patients in the example of hypertension (Meng et al., 2023) and type-2 diabetes mellitus (Verma et al., 2022). Meta-analyses provide evidence that health coaching reduces both disability and pain in people with chronic low back pain and reduces disability in people with knee osteoarthritis (Prior et al., 2023).

Health coaching is a client-centered approach to engaging users in setting personal goals to achieve positive health behavior changes that lead to improvements in self-care (Clason et al., 2023). Coaching is consistent with Rogers’s non-directive therapy theory assumed that “the capacity of the individual to reorganize his attitudes and behavior in ways not determined by external factors nor by previous elements in his own experience, but determined by his own insight into those factors, is an impressive capacity” (Rogers, 1946). These are realized by the holistic competencies of health coaching: the spirit of health coaching relationship and 50/50 patient/provider partnership in health; patient/client engagement through motivational interviewing and empathy; guiding the agenda and goal setting; communication style; cultural competence; active listening, mindfulness; facilitating behavior change; evidence-based practice interventions for wellness, prevention, and chronic health conditions (Huffman, 2016).

Coaching has the potential to reduce the treatment burden for people with chronic disorders and multiple long-term conditions (Matthews et al., 2023), including chronic fatigue, and facilitate the patient-centered care pathway (Gartner et al., 2022). In light of the strengthening of multidisciplinary team building, the coach acts as a complementary agent of the integrative approach, an implementer of team coaching, and a professional supporter of care providers (Körner et al., 2017, 2018; Rosen et al., 2022; Stephany et al., 2023).

In this article, the authors focused on the problems in patients with chronic diseases and multiple long-term conditions, with the example of chronic fatigue (CFS and ME/CFS), which often has a long time to diagnose, while post-diagnostic treatment is usually symptomatic and generalized. In these circumstances, an integrative approach from the diagnostic stage can both speed up the setting of diagnosis and improve the outcomes of treatment. The scientific literature points to the potential of coaching to strengthen the success factors of integrative care: a holistic approach, multidisciplinary team, and patient engagement. The authors underline the importance of further research on the perspective of coaching in integrated care.

## Data availability statement

The original contributions presented in the study are included in the article/supplementary material, further inquiries can be directed to the corresponding author.

## Author contributions

DA, MS, AK, ZN-K, UB, and MM: conceptualization and writing – review and editing. DA and MS: methodology. MS, AK, ZN-K, UB, and MM: validation. DA: formal analysis, investigation, writing – original draft preparation, and visualization. MM: supervision. All authors contributed to the article and approved the submitted version.

## Funding

The research was supported by the Horizon 2020 Project/Agreement No. 952376 “Reducing networking gaps between Riga Stradins University (RSU) and internationally leading counterparts in viral infection-induced autoimmunity research (VirA)” and Latvian Council of Science FARP Grant Number: LZP-2019/1-0380, “Selection of biomarkers in ME/CFS for patient stratification and treatment surveillance/optimization.”

## Conflict of interest

The authors declare that the research was conducted in the absence of any commercial or financial relationships that could be construed as a potential conflict of interest.

## Publisher’s note

All claims expressed in this article are solely those of the authors and do not necessarily represent those of their affiliated organizations, or those of the publisher, the editors and the reviewers. Any product that may be evaluated in this article, or claim that may be made by its manufacturer, is not guaranteed or endorsed by the publisher.
